# Discrimination of three *Ephedra* species and their geographical origins based on multi-element fingerprinting by inductively coupled plasma mass spectrometry

**DOI:** 10.1038/s41598-018-28558-9

**Published:** 2018-07-06

**Authors:** Xiaofang Ma, Lingling Fan, Fuying Mao, Yunsheng Zhao, Yonggang Yan, Hongling Tian, Rui Xu, Yanqun Peng, Hong Sui

**Affiliations:** 1 Ningxia Medical University Pharmacy College, Yinchuan, 750004 Ningxia China; 2 Ningxia Research Center of Modern Hui Medicine Engineering and Technology, Yinchuan, 750004 Ningxia China; 3 Key Laboratory of Hui Ethnic Medicine Modernization, Ministry of Education, Yinchuan, 750004 Ningxia China; 4 Shaanxi University of Chinese Medicine, Pharmacy College, Xianyang, 712046 Shaanxi China; 5 Institute of Industrial Crop Research, Shanxi Academy of Agricultural Sciences, Fenyang, 032200 Shanxi China

## Abstract

Discrimination of species and geographical origins of traditional Chinese medicine (TCM) is essential to prevent adulteration and inferior problems. We studied *Ephedra sinica* Stapf, *Ephedra intermedia* Schrenk et C.A.Mey. and *Ephedra przewalskii* Bge. to investigate the relationship between inorganic element content and these three species and their geographical origins. 38 elemental fingerprints from six major Ephedra-producing regions, namely, Inner Mongolia, Ningxia, Gansu, Shanxi, Shaanxi, and Sinkiang, were determined to evaluate the importance of inorganic elements to three species and their geographical origins. The contents of 15 elements, namely, N, P, K, S, Ca, Mg, Fe, Mn, Na, Cl, Sr, Cu, Zn, B, and Mo, of *Ephedra* samples were measured using inductively coupled plasma mass spectroscopy. Elemental contents were used as chemical indicators to classify species and origins of *Ephedra* samples using a radar plot and multivariate data analysis, including hierarchical cluster analysis (HCA), principal component analysis (PCA), and discriminant analysis (DA). *Ephedra* samples from different species and geographical origins could be differentiated. This study showed that inorganic elemental fingerprint combined with multivariate statistical analysis is a promising tool for distinguishing three *Ephedra* species and their geographical origins, and this strategy might be an effective method for authenticity discrimination of TCM.

## Introduction

Ephedrae herba (Mahuang) is a well-known traditional Chinese medicine (TCM) derived from the dried herbaceous stem of *Ephedra sinica* Stapf (*E. sinica*), *Ephedra intermedia* Schrenk et C.A.Mey.(*E. intermedia*), and *Ephedra equisetina* Bge^1^. It has been used for more than 5000 years in China^2^. Its main active ingredients are alkaloids, such as ephedrine and pseudoephedrine^3,4^. Flavonoids^5^, polysaccharides^6^, phenolic compounds^7^, and proanthocyanidins^8^, are also its active constituents. Mahuang relieves asthma and promotes diuresis and sweat. In modern TCM, it has been used to treat rheums, asthma, fever, rheumatoid arthritis^9^, and cough with dyspnea.

*Ephedra* is widely distributed in China, except in the lower reaches of the Yangtze River and the Pearl River Basin, and is especially common in the arid and semiarid regions of the northwest territories of China, such as Ningxia, Inner Mongolia, Sinkiang, and Gansu provinces^10,11^.

The market demand of Mahuang is strong, and its wild resources have been severely reduced by excessive harvesting^12^. Many *Ephedra* plants are morphologically similar, making their identification based on morphology very difficult, and some adulterants of *Ephedra* species are confused with the medicinal plants. The number of poor-quality Mahuang is increasing in the market, and the inferior medicinal materials mainly come from non-genuine production areas and non-medicinal *Ephedra* species, such as *Ephedra przewalskii* Stapf (*E. przewalskii*), which is easily confused with the medicinal *Ephedra* plants. *E. intermedia* and *E. przewalskii* both have two-lobed or three-lobed leaves^13^, which increases the difficulty in distinguishing them. The discrimination of species and geographical origins of medicinal *Ephedra* plants is essential to prevent adulteration and inferior problems^14^. Medicinal materials from different geographical origins are difficult to distinguish for the naked eye. Therefore, the quality control of genuine TCM often relies on chemical analysis.

The current elemental fingerprint study differentiated crops based on element composition and multivariate statistical analysis^15^. However, few fingerprints were reported on the inorganic element discrimination of *Ephedra*^16^. Inorganic elements contribute to the medicinal quality of TCM^17^. Some inorganic elements play important roles in the formation of active components, which are responsible the curative properties^18,19^. Therefore, constructing elemental fingerprints is useful in identifying the geographical origin and species of TCM.

The study on inorganic elements is very little in Mahuang, and the 15 inorganic elements of N, P, K, S, Ca, Mg, Fe, Mn, Na, Cl, Sr, Cu, Zn, B, and Mo are indispensable and irreplaceable for plant growth, therefore, these elements were selected in this study. According to the Chinese Pharmacopoeia^1^, Mahuang, as a traditional Chinese medicine, should be collected in the autumn growth stage. So the effects of the growth phase on these elements were the same for Mahuang, which increased the reliability for research.

In this study, we determined the 15 inorganic elements in *E. sinica*, *E. intermedia*, and *E. przewalskii* using ICP-MS, investigated the elemental compositions, discriminated three *Ephedra* plants from different regions through multivariate analysis, and further established a reliable method for differentiating the three *Ephedra* species. Results showed that the combination of inorganic elemental fingerprint with multivariate statistical analysis is a valuable tool to discriminate the geographical origins of Mahuang.

## Results and Discussion

### Method validation

15 elements were measured by external calibration using the ICP-MS elemental standard substances. Slopes of calibration curves were sensitive, and their correlation coeffcients all reached above 0.9995. The relative standard deviation (RSD), which stood for the precision, ranged from 0.02% to 2.6%. Withinday repeatability was <3.4%. The limit of detection (LOD) values were determined by using signal-to-noise ratios of 3:1, and Tables S1 showed the LODs determined in digestion solutions of plants samples for ICP-MS. The recoveries from plant sample S-NX3 ranged from 94% to 117% for ICP-MS.

### Element contents of samples

The contents of elements are shown in Supplementary Table S1. N content was the highest among the 15 elements. The sample of S-NM2 contained the lowest N content, and I-GS11 accumulated the highest N content, ranging extensively from 12523.58 to 34283.81 mg/kg. Mo content was the lowest among the 15 elements. The lowest and highest Mo contents occurred in S-NM2 and I-GS8, respectively, with contents varying from 0.59 to 1.54 mg/kg. Differences in element contents are due to different growth habits. The element contents of plants are affected by several factors, including plant characteristics (e.g., biological status, species), environmental conditions (e.g., humidity, temperature), and soil characteristics (e.g., pH, mineral composition)^20^.

### Construction of inorganic elemental fingerprint

**Figure 1:**
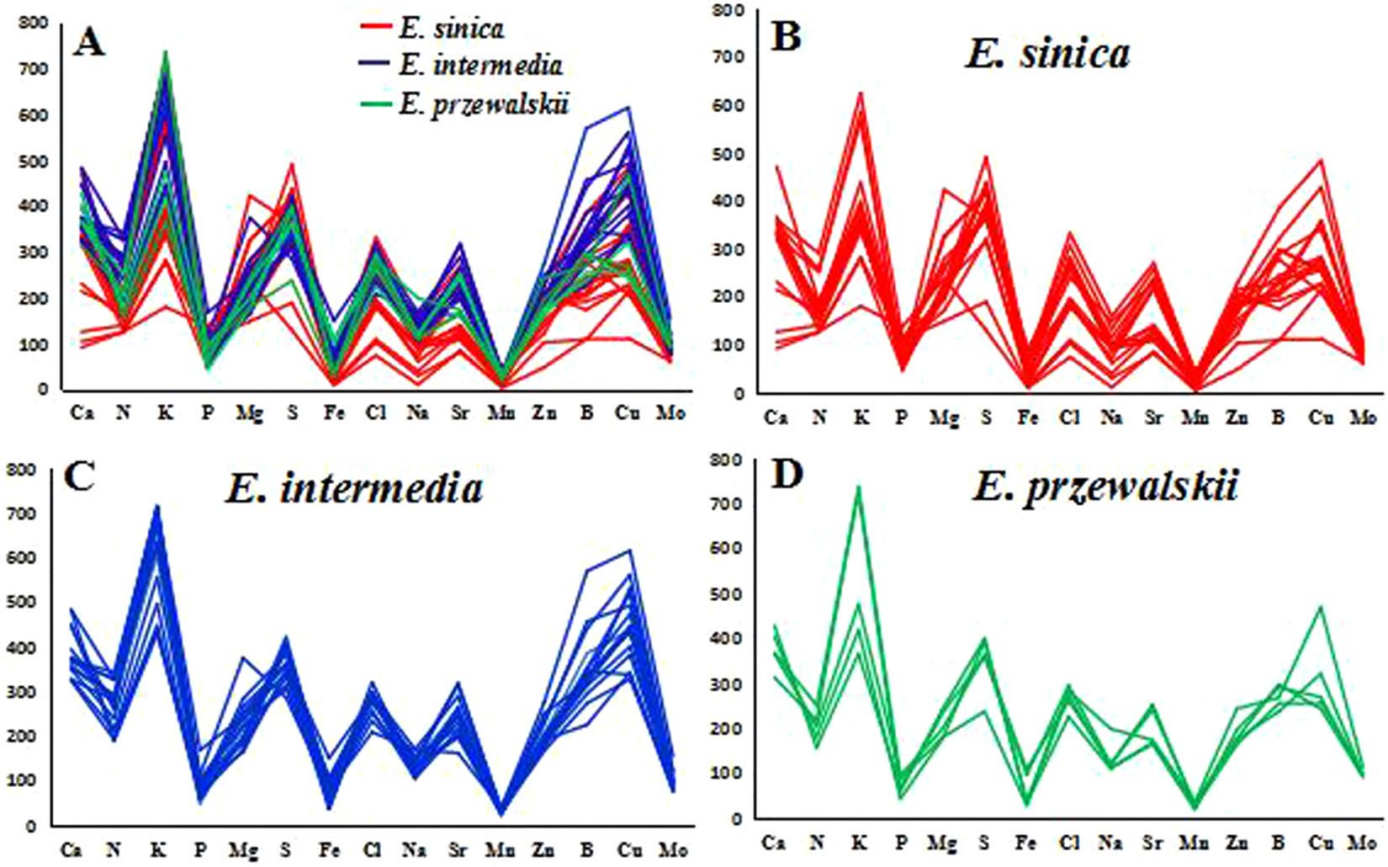
The element fingerprint common character of *Ephedra* samples. (**A**) Fingerprint character of elements average contents from three *Ephedra* species; (**B**) Elemental fingerprint character of *E. sinica* from 17 different origins; (**C**) Elemental fingerprint character of *E. intermedia* from 16 different origins. (**D**) Elemental fingerprint character of *E. przewalskii* from 5 different origins. The contents of several elements were narrowed or expanded to the same order of magnitude: Cu and Mo were expanded 100-fold; B and Zn were expanded ten fold; Ca, Mg, P, K, Fe, and S were reduced ten fold; and N was reduced 100-fold.

To demonstrate the distribution rules of the elemental contents, we used the 15 elements to construct an inorganic elemental fingerprint based on the results of quantitative detection through ICP-MS. For convenience, the contents of several elements were narrowed or expanded to the same order of magnitude. Cu and Mo were expanded 100-fold; B and Zn were expanded tenfold; Ca, Mg, P, K, Fe, and S were reduced tenfold; and N was reduced 100-fold)^21^. The 15 elements of the 38 *Ephedra* samples were drawn in one plot for comparison, as shown in Fig. 1.

Clearly, the fingerprints of three *Ephedra* species exhibited a common character based on the elements average contents (Fig. 1A). All *Ephedra* samples also showed a similar peak (Fig. 1B–D), the content of an individual element was always maintained within a certain range^22^ for the samples of different geographical origins. However each element content was different in these fingerprints, and the differences in the elemental contents were related to the diverse origins and species of *Ephedra*. The elemental contents order of 3 *Ephedra* species were *E. intermedia* > *E. przewalskii* > *E. sinica* except for P, S, Cl, and Mo. Especially for Ca, N, K, Sr, B and Cu, the elemental contents in *E. intermedia* were significantly higher than those in *E. sinica*. Therefore, the elemental fingerprints of *Ephedra* samples distinguished between *E. intermedia* and *E. sinica*. Nevertheless, the elemental fingerprints of *Ephedra* samples difficultly distinguish *E. przewalskii* from *E. intermedia* and *E. sinica*, and can’t differentiate the *Ephedra* samples from different geographical origins, so the hierarchical cluster analysis (HCA) was subsequently performed.

### Sample distribution according to HCA

**Figure 2:**
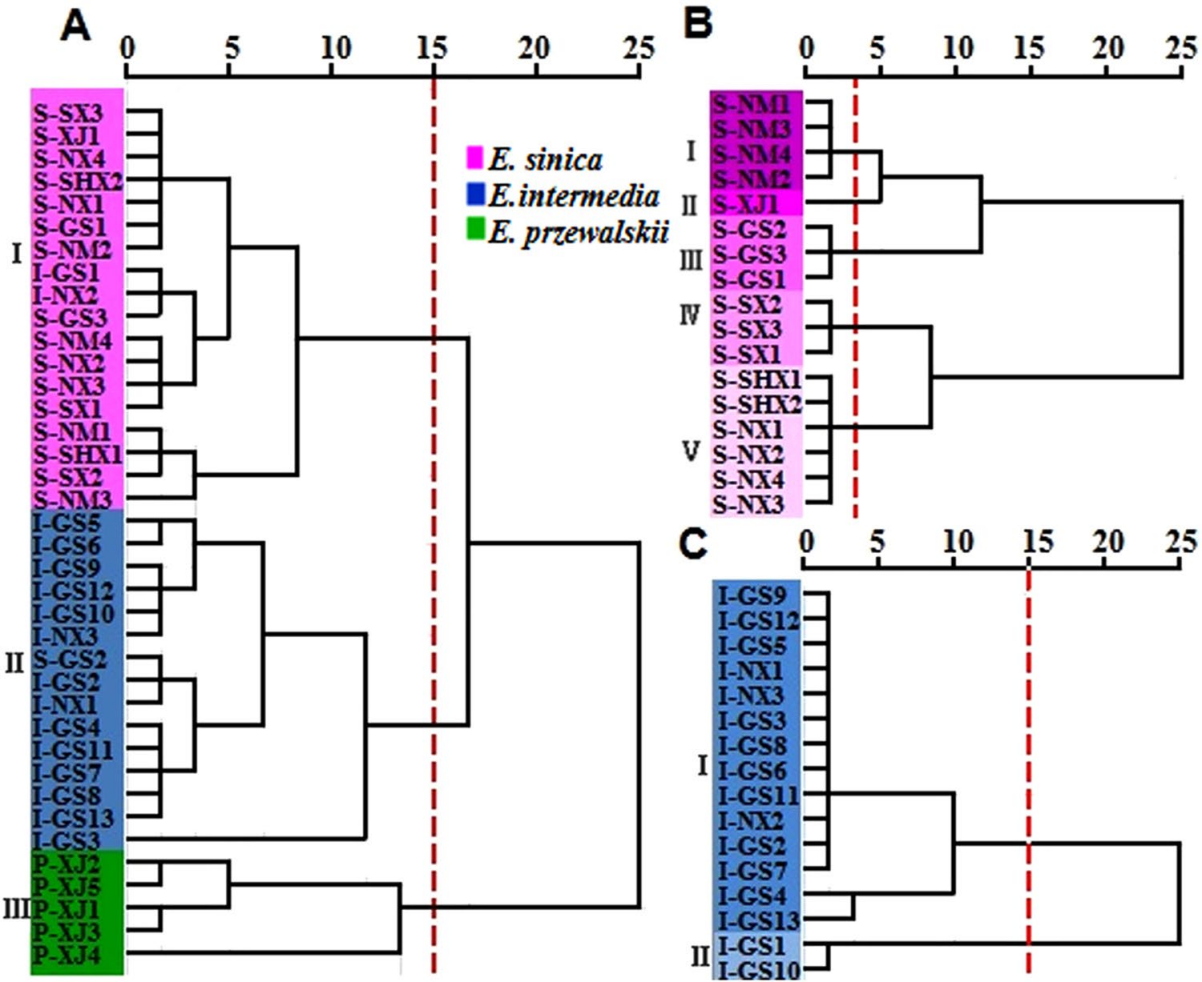
Dendrograms of hierarchical cluster analysis (HCA) based on 15 elements for *Ephedra* samples. (**A**) Showed 38 samples of three *Ephedra* species; (**B**) showed *E. sinica* samples from 17 different origins; (**C**) showed *E. intermedia* samples from 16 different origins. S-NM: *E. sinica* samples from Inner Mongolia; S-NX: *E. sinica* samples from Ningxia; S-GS: *E. sinica* samples from GanSu; S-SX: *E. sinica* samples from ShanXi; S-SHX: *E. sinica* samples from ShaanXi; S-XJ: *E. sinica* samples from Sinkiang; I-NX: *E. intermedia* samples from Ningxia; I-GS: *E. intermedia* samples from GanSu; P-XJ: *E. przewalskii* samples from Sinkiang.

HCA of the samples was performed to distinguish among the three *Ephedra* species in samples and the different origins of *E. sinica* and *E. intermedia* based on all the elemental data. *Ephedra* samples of different species were separated into three clusters based on the dendrogram cut at distance of 15 (Fig. 2A)^23^. The first cluster comprised sixteen *E. sinica* and two *E. intermedia* samples, the second cluster consisted of fourteen *E. intermedia* and one *E. sinica* samples, followed by a third cluster of *E. przewalskii*. The cluster results were in agreement with the actual species of *Ephedra* samples, especially for *E. przewalskii*, which was usually considered as a counterfeit herb. Thus, it can be completely separated from medicinal *Ephedra* (*E. sinica* and *E. intermedia*). These results implied that element information can be suitably utilized to classify *Ephedra* samples of different species.

The *E. sinica* samples from different origins were separated into five clusters based on a dendrogram cut at a distance of 15 (Fig. 2B). The first, second, third, fourth, and fifth clusters were composed of *E. sinica* samples from Inner Mongolia (4), Sinkiang (1), Gansu Province (3), Shanxi (3), and Shaanxi (2) and Ningxia (4), respectively.

*E. intermedia* samples from different origins were separated into two clusters (Fig. 2C). The first cluster was composed of fourteen samples, three of which were from Ningxia province and eleven from Gansu Province. The second cluster comprised two *E. intermedia* samples in Gansu Province. Eleven *E. intermedia* samples in Gansu province and three in Ningxia province clustered in one class, which might be due to the adjacency of the two provinces and the proximity of these fourteen sampling sites. The other two areas of Gansu province were in the second cluster mainly because the sampling points were in the Northwest direction and were far from the first fourteen sampling points.

It is difficult to identify the three *Ephedra* species and their geographical origins with the uniform criterion. The samples with low similarities are easily identified at high Euclidean distances. The samples with high similarities are not easily identified, and their discriminations are often at a low Euclidean distance. *E. sinica*, *E. intermedia*, and *E. przewalskii* are different species with low similarity, and are distinguished at Euclidean distance 15. *E. sinica* from different provinces are not easily identified because of their high intraspecific similarity, and they are distinguished at Euclidean distance 3.5 generally. *E. intermedia* are all from Gansu Province except adjacent Ningxia, and have high similarities. Two samples of them are identified at Euclidean distance 15, and the other samples are difficult to identify.

A slight overlap occurred between *E. sinica* and *E. intermedia* and the geographical origins of the samples probably because the geographical location was close and the ecological environment was similar^24^. Subsequently, a radar plot was used to further study the discrimination of the samples from different regions and various species.

### Distinguishing species and geographical origins of samples by radar plot

**Figure 3:**
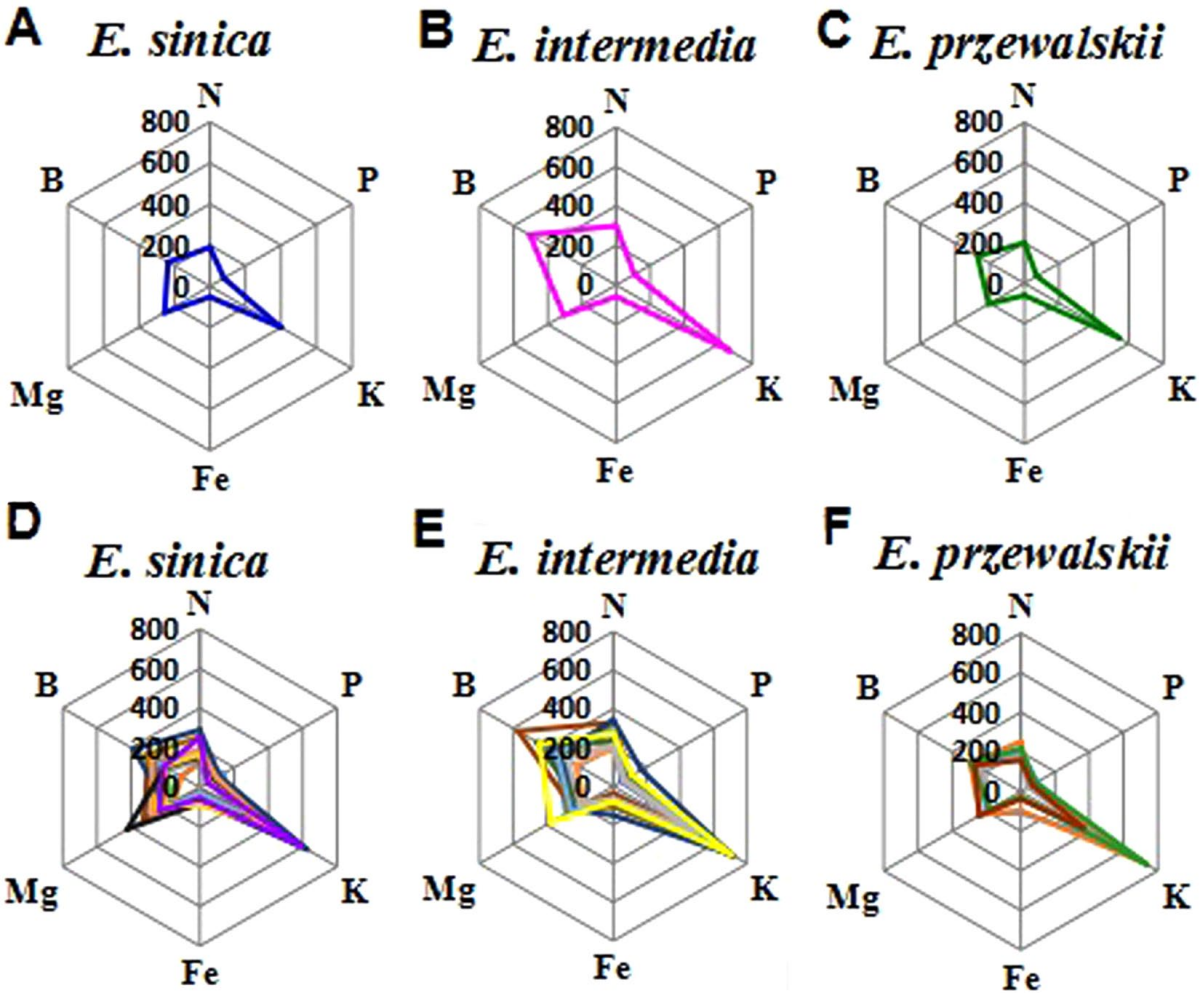
A radar plot showing the difference in species and geographical origins of *Ephedra* samples based on six elements (B, N, P, K, Mg, and Fe). (**A**) Showed the elements average contents of *E. sinica*; (**B**) showed the elements average contents of *E. intermedia*; (**C**) showed the elements average contents of *E. przewalskii*; (**D**) showed the elements contents of *E. sinica* from 17 different origins; (**E**) showed the elements contents of *E. intermedia* from 16 different origins; (**F**) showed the elements contents of *E. przewalskii* from 5 different origins.

To visually compare the results, we constructed a radar plot to classify the *Ephedra* samples. This method allows routine, simple, and rapid discrimination^25^. It was used for distinguishing the geographical origins and species of the *Ephedra* samples based on six elements, namely, N, K, Mg, P, Fe, and B. These elements were chosen because their relative standard deviation (RSD) were high in *Ephedra* samples. Figure 3A–C showed the distributions of the elemental patterns of three *Ephedra* species based on the mean content of the six elements. *E. sinica*, *E. intermedia* and *E. przewalskii* presented clearly different characteristic patterns and were easily distinguished. This finding corresponded with the HCA results. Radar plot analyses were performed for 17 *E. sinica*, 16 *E. intermedia* and 5 *E. przewalskii* samples collected from different regions of 6 provinces. Figure 3D–F show the differences in geographical origins of *E. sinica*, *E. intermedia*, and *E. przewalskii* samples, respectively. Some elements in one *Ephedra* species vary in different regions^26^. The B and Mg contents changed for *E. sinica* and *E. intermedia* samples from different sampling locations, whereas N and K contents revealed different patterns for *E. przewalskii* samples from five different areas. Therefore, this method illustrated the geographical origins of different samples from the same species. Figure 3D–F also illustrated that the same species in different samples exhibited similar elemental pattern characteristics, indicating that the samples from the same species were of similar genetic background. However, the visual chart from the radar plot analysis lacked the specific indexes to describe the exact differences, which decreased the confidence level of the results. Therefore, principal component analysis (PCA) was subsequently performed.

### Identification of species and geographical origins of samples by PCA

PCA was performed by factor analysis in SPSS software. PCA was implemented on element contents based on the 15 variables to classify the 38 *Ephedra* samples from different species and geographical origins. Table 1 showed the vectors and cumulative contribution of variance of the first four PCs (PC1–4). A four-factor model (the first four PCs with eigenvalues > 1) can explain 81.294% of the total variability in the original data^21^. The PC1, PC2, PC3, and PC4 contributed 53.452%, 12.633%, 8.423%, and 6.786% of the total variance, respectively. The first four PCs demonstrated that the N, K, Cl, Na, B, Mn, and Cu weights were high in PC1; P content loaded highly in PC2; and Mg played a major feature content in PC3 and PC4. The 81.294% contribution varied from PC1 to PC4. Therefore, the elements N, K, B, Mn, Na, Cu, Cl, P, and Mg were regarded as the characteristic elements in the *Ephedra* samples. These elements may be treated as the most powerful referents of *Ephedra* samples.

**Table 1:** The vectors and cumulative contribution of variance of the first four principal components.

Items	Principal component			
	1	2	3	4
N	0.851	0.357	−0.143	−0.088
K	0.816	0.307	−0.207	−0.058
S	0.342	−0.716	−0.005	−0.002
Ca	0.733	−0.422	−0.097	0.154
Mg	0.040	−0.197	0.569	0.745
P	−0.038	0.851	0.202	0.165
Fe	0.661	0.187	−0.464	0.421
Cl	0.835	−0.241	−0.194	−0.047
Sr	0.799	0.081	−0.305	0.357
Na	0.888	−0.188	0.072	−0.116
B	0.829	0.050	0.313	−0.127
Mn	0.880	−0.052	−0.061	−0.012
Zn	0.762	−0.105	0.368	−0.203
Cu	0.883	0.211	0.190	−0.143
Mo	0.766	0.153	0.449	−0.050
Variance (%)	53.452	12.633	8.423	6.786
Cumulative variance (%)	53.452	66.085	74.508	81.294

**Figure 4:**
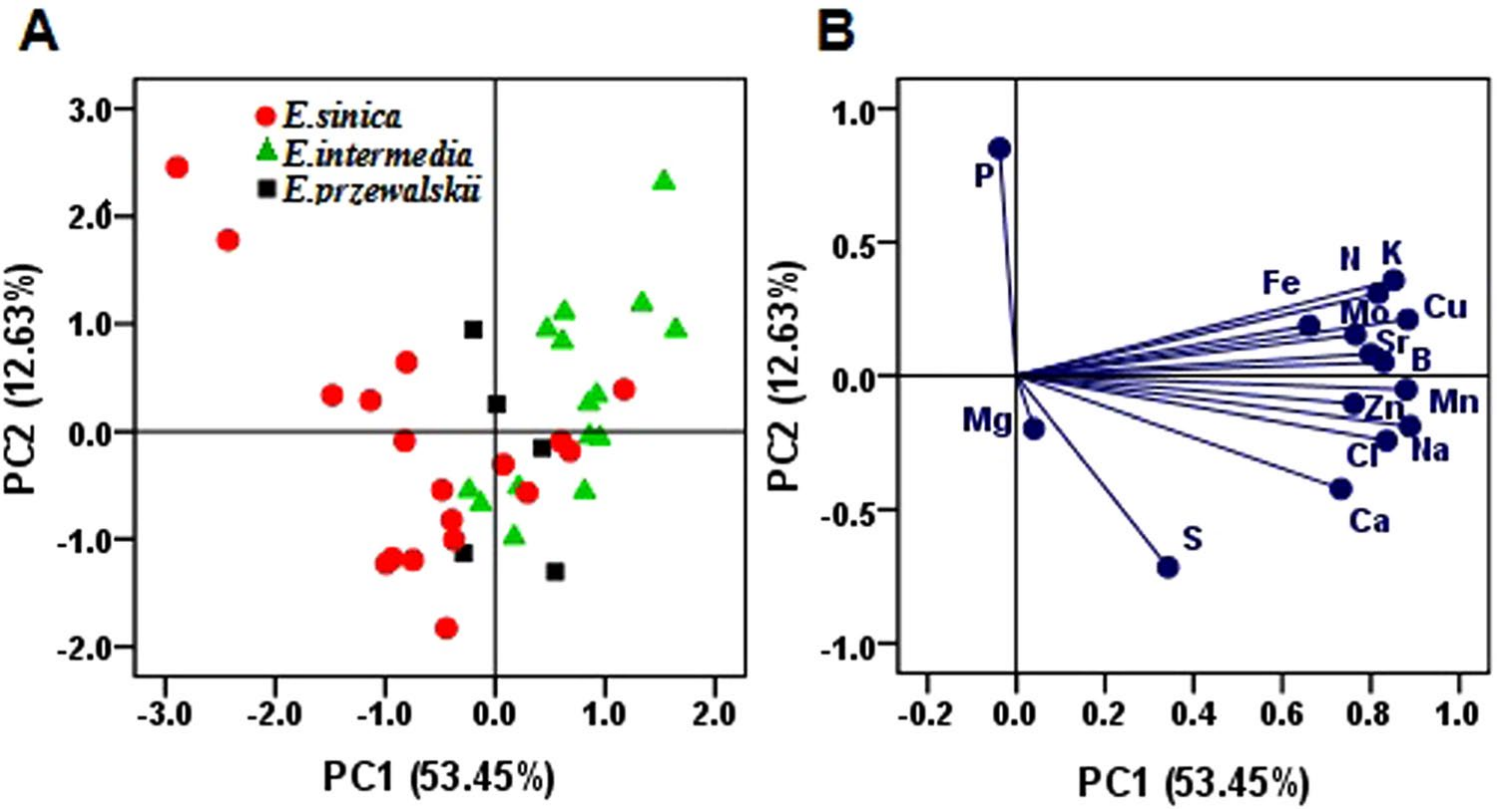
Principal component analysis (PCA) based on 15 elements in *Ephedra* samples. Panel (A,B) illustrate the score plot and the corresponding loading plot of 38 *Ephedra* samples from different species. Grouping according to species was shown by principle components 1 and 2, which explained 53.45% and 12.63% of the variance, respectively.

Figure 4A showed the score plot of the first two PCs which accounted for 66.08% of total variance in raw data (PC1 = 53.45%, PC2 = 12.63%). A basic separation between *E. sinica* and *E. intermedia* could be observed, with *E. przewalskii* at the middle^27^. The corresponding loading plot (Fig. 4B) described the variables related to the separation, and the elements N, K, B, Mn, Na, Cu, Cl, and P controlled the discrimination of the three *Ephedra* species.

The PCA score plots (Supplementary Fig. S1A) illustrated a separation pattern of the *E. sinica* samples, and the corresponding loading plot (Supplementary Fig. S1B) showed that K, Cu, Sr, Na, and Cl influenced the separation of *E. sinica* from different regions. The samples from six growing areas could be approximately separated. Table 2 indicates that the first three PCs (PC1–3), which have eigenvalues >1, explained 79.595% of the total variability among the 15 variables in the original data of *E. sinica* samples, where PC1, PC2, and PC3 contributed 60.15%, 12.46%, and 6.99% of the total variance^28^. Supplementary Fig. S1C displays the separation pattern of the *E. intermedia* samples, whereas Supplementary Fig. S1D presents the corresponding loading plot, illustrating the variables related to the separation. N, Mn, Cu, Mo, and Zn affected the discrimination of *E. intermedia* samples. The plot of *E. intermedia* samples was defined by the PC1 and PC2, which explained 41.38% and 14.86% of the variance, respectively. The *E. intermedia* samples from Ningxia and Ganshu were distinguished.

**Table 2:** Cumulative contribution of variance of the principal components.

Component	*E. sinica*			Component	*E. intermedia*		
	Total	% of Variance	Cumulative %		Total	% of Variance	Cumulative %
1	9.023	60.153	60.153	1	6.206	41.375	41.375
2	1.868	12.455	72.608	2	2.228	14.855	56.229
3	1.048	6.988	79.595	3	1.607	10.712	66.942
				4	1.519	10.126	77.068
				5	1.106	7.374	84.442

Clearly, the PCA results obtained were preliminary, and no obvious separation between *Ephedra* samples of different species and regions were observed in the PCA score plot according to element contents. Some overlaps were observed in the three *Ephedra* samples. Therefore, discriminant analysis (DA) was used to improve the separation and to further identify the authenticity of the samples.

### Discrimination of species and geographical origins of samples based on DA

DA was applied to classify groups of *Ephedra* samples from different species and geographical origins based on element contents. The calculation was carried out using nine variables^25^, namely, Ca, S, P, Zn, Mg, Fe, Mn, Na, and B contents, to classify the three *Ephedra* species, and the other elements cannot be used for the discriminant function due to their insignificant discrimination effects.

**Figure 5:**
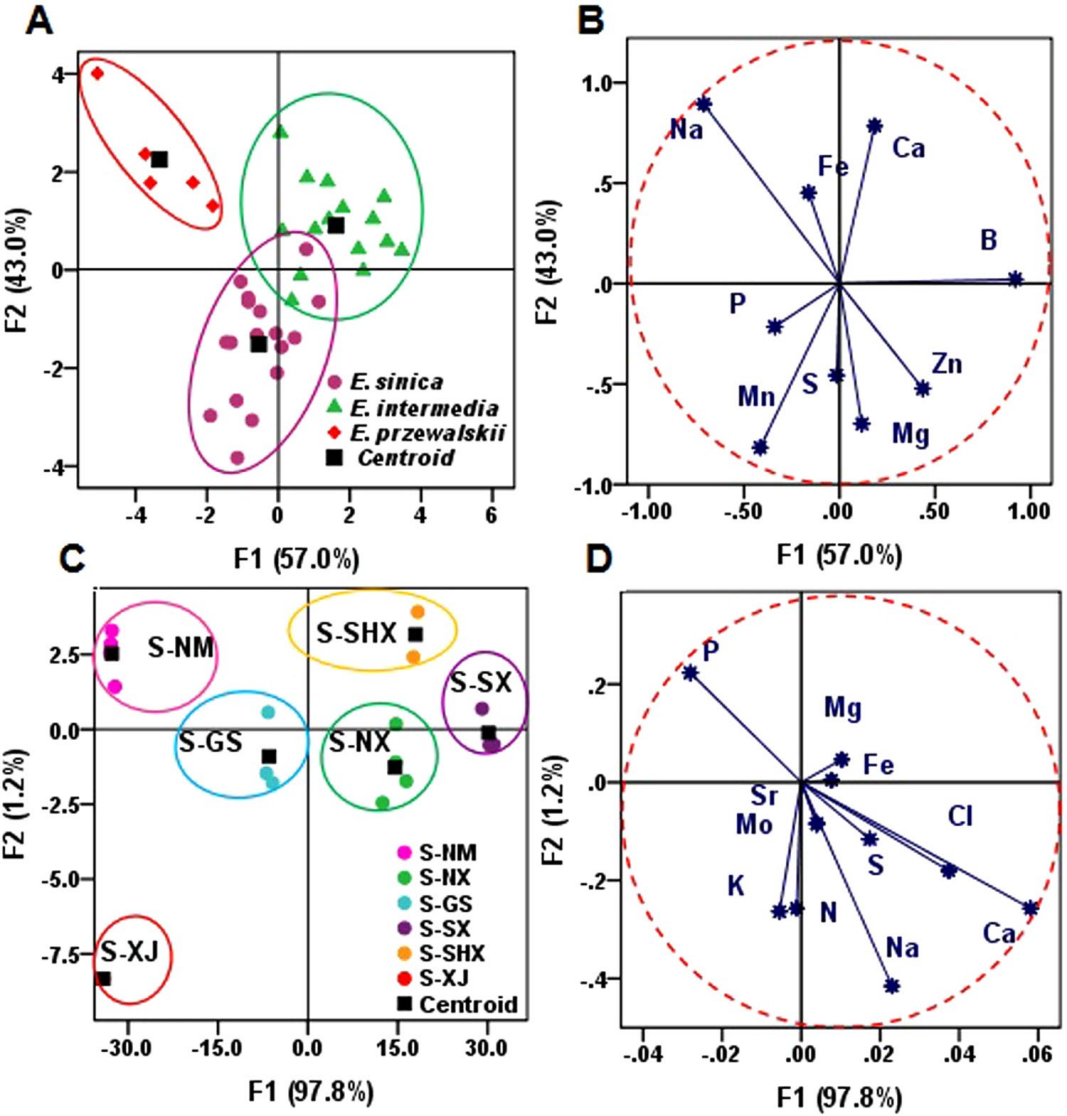
Discrimination analysis for *Ephedra* samples from different species and regions (**A**) The discrimination of three species of *Ephedra* samples (*E. intermedia*, *E. sinica, E. przewalskii*); (**B**) Correlation chart between the selected variables and the discriminant functions for the 38 *Ephedra* samples; (**C**) The discrimination of *E. sinica* samples from different geographical regions; (**D**) Correlation chart between the selected variables and the discriminant functions for *E. sinica* samples.

Figure 5A shows the distribution patterns of the three *Ephedra* species in the plot defined by the discriminant functions. The variations between groups were explicated by the discriminant functions 1 (57.0%) and 2 (43.0%). Samples of *E. przewalskii* were completely differentiated from that of *E. sinica* and *E. intermedia*. Figure 5B represents the load correlation of the selected elements from all samples in the plane designed by the first two discriminant functions (F1 and F2). F1 contributed 57.0% of the variance, affording the primary species identification in all of the samples, and was positively correlated with the contents of Ca, Mg, Zn and B. F2 provided 43.0% of the variance and positively correlated with Na, Ca, and Fe contents^29^. Zn, B, Ca, and Na were regarded as the most useful variables for species identification in all of the samples according to the correlation between Fig. 4A,B. This study showed that the samples from different species were plotted in different spaces.

DA was further implemented based on the contents of eleven elements, namely, P, Mg, Sr, Fe, Mo, K, N, Na, S, Ca, and Cl of *E. sinica* samples from different regions, as shown in Fig. 5C. The distribution pattern of *E. sinica* samples was defined by the discriminant functions 1 and 2, and the two functions explained 97.8% and 1.2% of the variance, respectively. The *E. sinica* samples from different regions were completely separated. By using DA of *E. sinica* samples, we were able to distinguish the different samples from Inner Mongolia, Ningxia, Gansu, Shanxi, Shaanxi, and Sinkiang of China and observed the projections, which were located in different dimensions of the chart. F1 represented 97.8% of the variance, provided the main separation between the six provinces, and exhibited a strongly positive correlation with Na, Ca, Cl, and S contents. F2 accounted for 1.2% of the variance and was positively correlated with Mg and P contents (Fig. 5D). Therefore, the main separators between the six provinces were Na, Ca, Cl, S, Mg, and P.

DA was performed based on the contents of fourteen elements, namely, N, P, K, S, Ca, Mg, Fe, Mn, Na, Cl, Sr, Cu, Zn, and B, of *E. intermedia* samples from different regions. These elements were chosen as the variables of the discriminant function because of their significant influences. The distribution pattern of *E. intermedia* samples was defined by the discriminant function 1, which explained 100.0% of the variance. The *E. intermedia* samples from different regions were completely separated.

To check the reliability of the DA classification model, we performed a cross-validation procedure to calculate the classification and probability of the *Ephedra* samples^24^. Table 3 displays the cross-validation results together with the classification of *Ephedra* samples by the DA model. The results showed that 92.1% of all *Ephedra* samples were correctly classified, among which 100% of *E. przewalskii* were correctly differentiated from the *E. sinica* and *E. intermedia* samples, and 100% of 17 *E. sinica* and 16 *E. intermedia* samples from different growing provinces were correctly authenticated^23^. These results were parallel with those of previous studies. Therefore, multi-element analysis with chemometrics method is a promising fingerprinting analytical strategy for classifying *Ephedra* samples.

**Table 3:** Classification Results of *Ephedra* samples using discriminant analysis.

Origin	Assigned species for *Ephedra* samples with 9 variables							
	*E. sinica*		*E. intermedia*		*E. przewalskii*		Total	Correct (%)
*E. sinica*	15		2		0		17	88.2
*E. intermedia*	1		15		0		16	93.8
*E. przewalskii*	0		0		5		5	100.0
Total	16		17		5		38	92.1
Origin	Assigned origin for *E. sinica* samples with 11 variables							
	S-NM	S-NX	S-GS	S-SX	S-SHX	S-XJ	Total	Correct (%)
S-NM	4	0	0	0	0	0	4	100.0
S-NX	0	4	0	0	0	0	4	100.0
S-GS	0	0	3	0	0	0	3	100.0
S-SX	0	0	0	3	0	0	3	100.0
S-SHX	0	0	0	0	2	0	2	100.0
S-XJ	0	0	0	0	0	1	1	100.0
Total	4	4	3	3	2	1	17	100.0
Origin	Assigned species for *E. intermedia* samples with 14 variables							
	S-NX			S-GS			Total	Correct (%)
S-NX	3			0			3	100
S-GS	0			13			13	100
Total	3			13			16	100

## Materials and Methods

### Materials

In this study, the herbaceous stems of *E. sinica, E. intermedia* and *E. przewalskii* were collected from 38 producing areas in six provinces of China, namely, Inner Mongolia, Ningxia, Gansu, Shanxi, Shaanxi, and Sinkiang, in September and October 2012 (Supplementary Table S2 and Fig. S2). In each producing area, 5 sampling sites were selected to collect 500 g plant samlpes (100 g for one sampling site), the distance between two sampling locations were above 200 meters to ensure the samples to be representative.

### Sample preparation

The herbaceous stems of the samples were rinsed with the deionized water and dried at 105 °C. The dried samples were ground into fine powders of 100mesh and stored in plastic bags at room temperature before analysis. The microwave assisted digestion was processed for the Ephedra samples (in triplicate) by a Mars-6 Microwave System (CEM Co., Ltd, U.S.), and the following project was used: About 1.000 g sample was precisely weighed inside teflon digestion vessels, and digested in the liquid of 3.0 mL H_2_O_2_ and 5.0 mL HNO_3_ with a three-step procedures (first: 120°C/20 min; second: 160 °C/20 min; third: 180 °C/45 min). Then the digested solution was evaporated to dryness on a hot plate with 150 °C. The digestions were cooled to room temperature, and diluted with deionized water to 10 mL in a volumetric flask.

### ICP-MS measurements

N, P, K, S, Ca, Mg, Fe, Mn, Na, Cl, Sr, Cu, Zn, B, and Mo were determined using ICP-MS (NexION 300D, PerkinElmer Instrument Co., U.S.) following the modified JIS K0133-2007 method(Japanese Industrial Standards Committee 2007) in Beijing ZKLH Analyzing & Testing Center. The parameters of the instrument were optimized as follows: sample uptake of 1.0 mL/min, radio frequency power of 1600 W, carrier gas flow rate of 1 L/min, plasma gas flow rate of 18.0 L/min, auxiliary flow rate of 1.20 L/min, dwell time of 50.0 ms, scan time of 20 s,and integral time of 1 s.

### Statistical analysis

Excel 2010 and SPSS 21.0 software were used for data analysis. The *Ephedra* samples were discriminated by radar plot analysis, element fingerprint analysis, and multi-element analysis, including principal component analysis (PCA), hierarchical cluster analysis (HCA)^21^, and discriminant analysis (DA). Elemental fingerprint, HCA, PCA and DA were based on all of the 15 elements from objects of study, respectively. The radar plot analysis was based on the six elements of N, K, Mg, P, Fe, and B with high relative standard deviation. PCA was used to primarily evaluate between-class similarity and achieve dimension reduction. HCA was a classification procedure which involved the measurement of similarity between the objects to be clustered. The amalgamation rule was based on Ward’s method, and the squared Euclidean distance was performed for the similarity measurement. Samples were grouped in clusters based on their distance and similarities^30^. DA classified the maximum variance between groups and minimum variance within groups by creating new variables, maximizing the variance between categories and minimizing the variance within categories. This technique is an effective and well-known method for classification^23^.

## Conclusion

In this paper, we measured the contents of 15 plant essential elements in *E. sinica*, *E. intermedia* and *E. przewalskii*, built their element fingerprints, investigated the mineral element ability to differentiate the three species and their geographical origins with chemometrics method. The radar plot and HCA clearly ditinguished the three *Ephedra* species, DA completely differentiated *E. przewalskii* from *E. sinica* and *E. intermedia*, correctly classified the geographical origins of *E. sinica* and *E. intermedia* samples. The combination of the element fingerprints and multivariate statistical techniques could be a practical tool for identifying the geographical origins and species of Mahuang.

### Data Availability

All data generated or analysed during this study are included in this published article (and its Supplementary Information files).

## Electronic supplementary material

Supplementary Information
