# Supplementary material for: Discrimination of three Ephedra species and their geographical origins based on multi-element fingerprinting by inductively coupled plasma mass spectrometry
Source: Sci Rep. 2018 Jul 6;8:10271. doi: 10.1038/s41598-018-28558-9 (PMC6035214; doi:10.1038/s41598-018-28558-9)
Supplement: Supplementary file 1 — Supplementary Information [file 41598_2018_28558_MOESM1_ESM.pdf]

Discrimination of three *Ephedra* species and their geographical origins based on multi-element fingerprinting by inductively coupled plasma mass spectrometry

Xiaofang Ma[1], Lingling Fan[1], Fuying Mao[1,2], Yunsheng Zhao[1,2,3]*, Yonggang Yan[4], Hongling Tian[5], Rui Xu[1], Yanqun Peng[1], Hong Sui[1,2]

1 Ningxia Medical University Pharmacy College, Yinchuan 750004, Ningxia, China

2 Ningxia Research Center of Modern Hui Medicine Engineering and Technology, Yinchuan 750004, Ningxia, China

3 Key Laboratory of Hui Ethnic Medicine Modernization, Ministry of Education, Yinchuan 750004, Ningxia, China

4 Shaanxi University of Chinese Medicine, Pharmacy College, Xianyang 712046, Shaanxi, China

5 Institute of Industrial Crop Research, Shanxi Academy of Agricultural Sciences, Fenyang 032200, Shanxi, China

*Corresponding Author: Yunsheng Zhao (zwhjzs@126.com)

Tel: +86-13619501878

Fax: +86-09516980193

Pharmacy College, Ningxia Medical University, No.1160 Shengli South Street, Xingqing District Yinchuan of China

Table S1. Elemental contents (mg kg $^{-1}$) in the *Ephedra* samples from different regions

LOD	Ca	N	K	P	Mg	S	Fe
	0.004	0.01	0.001	0.002	0.004	0.01	0.002
**S-NM1**	916.43±22.57	12674.39±24.61	1798.69±32.25	1370.41±15.41	2369.99±10.01	1294.90±5.09	100.57±3.56
**S-NM2**	1047.60±12.59	12523.58±13.57	2810.56±22.03	1092.29±7.71	1488.13±21.13	1890.27±9.73	120.27±2.27
**S-NM3**	1259.11±19.11	14035.03±26.03	3361.83±24.28	1068.45±11.54	2535.34±0.34	4193.58±6.42	167.13±2.13
**S-NM4**	2138.18±8.17	17225.55±17.54	4373.33±21.84	1022.76±21.24	2645.57±11.57	3619.69±16.31	760.71±4.28
**S-NX1**	3187.47±20.47	12895.00±5.00	2796.88±23.59	484.08±6.08	2223.01±10.98	4903.40±9.60	620.38±1.62
**S-NX2**	3319.03±1.97	18639.95±15.04	3627.49±14.48	984.20±3.79	1708.60±1.40	3207.46±10.53	215.52±0.52
**S-NX3**	2351.81±13.87	14492.52±8.42	3314.61±31.86	739.75±16.75	2281.86±11.84	3390.29±7.26	286.56±3.59
**S-NX4**	3358.88±18.87	15091.58±10.42	3512.01±27.86	633.25±11.75	3251.84±16.84	4180.26±9.73	206.40±2.59
**S-GS1**	2309.26±9.26	15298.38±12.61	3897.24±12.02	915.45±16.54	2058.22±8.77	3944.55±10.44	443.52±1.48
**S-GS2**	3529.95±15.05	25602.48±10.51	5650.18±20.87	702.76±4.75	1965.80±12.20	3806.45±6.55	743.89±1.10
**S-GS3**	3602.38±20.62	29009.41±13.59	6241.97±32.65	1150.93±7.92	1947.43±7.57	4346.19±7.80	675.68±2.31
**S-SX1**	3295.43±4.57	17584.78±15.22	3519.41±25.16	614.65±12.64	2250.72±9.27	3151.26±12.73	350.96±3.04
**S-SX2**	3284.36±13.64	17976.42±23.58	3523.78±33.86	709.65±9.64	2206.38±12.61	4376.49±10.51	260.99±2.01
**S-SX3**	4700.99±9.01	18890.55±19.45	3975.80±23.20	989.43±0.56	2792.54±7.46	3638.70±7.69	630.96±2.03
**S-SHX1**	3665.74±4.26	15812.47±20.53	3674.92±24.18	650.25±14.75	4225.56±19.44	3628.64±16.35	793.56±4.44
**S-SHX2**	3485.44±14.56	16632.28±17.72	3865.45±24.00	546.75±9.25	2039.29±14.71	3863.69±12.31	898.72±1.28
**S-XJ1**	3260.95±6.05	25160.22±16.77	5847.39±14.65	463.50±7.50	2313.56±20.43	3698.18±1.82	569.23±1.76
**I-GS1**	4816.93±16.81	18863.61±13.61	4284.02±30.83	782.21±12.21	2830.34±11.34	3821.97±28.02	365.03±9.02
**I-GS2**	4502.89±3.56	20996.08±51.08	4386.24±32.80	582.59±15.59	2204.64±14.64	3122.18±12.17	551.92±6.91
**I-GS3**	4445.75±13.92	25912.70±22.70	6722.28±36.20	684.71±6.71	1910.69±20.68	3944.90±11.09	976.84±9.83
**I-GS4**	3547.10±17.19	28136.77±36.76	6139.16±28.35	1050.94±27.05	1818.83±8.82	3311.91±11.90	887.47±19.46
**I-GS5**	3600.27±13.26	27821.80±9.79	6065.96±28.23	483.79±5.79	2232.74±11.73	3994.12±35.12	639.71±5.28
**I-GS6**	3267.25±11.36	27957.07±23.06	6797.40±24.75	985.15±18.14	2214.83±8.17	3061.43±16.42	665.46±11.54
**I-GS7**	3753.42±24.07	28605.13±13.87	6348.01±28.07	921.50±21.50	1635.11±9.88	3469.54±8.46	1056.13±12.13
**I-GS8**	3660.15±18.63	32868.37±13.37	6250.37±32.00	1013.41±12.41	2369.99±8.01	3424.01±31.98	376.53±20.53
**I-GS9**	3754.43±4.08	32504.09±5.09	7153.74±20.83	598.26±20.26	1976.08±9.07	4021.69±12.31	710.55±12.54
**I-GS10**	4830.98±0.67	32809.85±19.84	7125.22±20.46	694.03±16.03	2663.94±5.06	3577.02±10.98	1033.12±10.11
**I-GS11**	3698.63±20.74	34283.81±16.80	7067.77±32.51	1679.18±7.82	2425.10±9.90	3623.73±21.26	1497.56±19.56
**I-GS12**	3489.54±13.53	23995.40±28.40	5546.68±25.21	924.38±18.62	1923.99±21.00	4050.17±16.82	839.44±8.55
**I-GS13**	3537.13±8.87	29258.53±28.47	6709.87±18.99	710.13±12.87	2526.97±18.03	3589.59±11.59	784.63±5.37
**I-NX1**	3241.37±6.92	19221.48±7.51	4473.09±24.01	664.70±19.70	1690.22±12.21	4011.42±11.57	688.83±22.83
**I-NX2**	3276.22±9.22	21329.20±48.79	4957.04±32.25	590.33±23.33	1965.80±20.79	4210.67±23.32	634.32±10.68
**I-NX3**	3940.41±7.37	27189.28±9.72	7041.95±15.31	1125.83±13.82	3747.89±8.10	2853.22±17.22	788.06±21.05
**P-XJ1**	3654.79±11.99	20430.63±25.37	4748.21±25.20	953.70±8.70	1984.18±17.17	3632.58±12.42	1086.10±19.09
**P-XJ2**	3677.87±20.98	25112.38±22.37	7236.57±40.41	614.70±14.70	2340.10±9.09	3590.43±12.42	961.43±7.43
**P-XJ3**	4033.72±21.28	17999.43±0.56	4183.18±26.46	435.15±17.85	1767.39±11.39	3931.75±13.25	342.70±18.70
**P-XJ4**	3126.61±0.60	21694.84±16.84	7365.34±11.95	871.20±4.20	1795.25±8.25	2375.09±8.09	283.79±7.79
**P-XJ5**	4276.29±18.72	15704.29±8.29	3649.78±35.94	667.35±10.64	2425.10±8.90	3994.31±3.68	371.03±15.03

Table S1. Cont. Elemental contents (mg kg $^{-1}$) in the *Ephedra* samples from different regions

LOD	Cl	Na	Sr	Mn	Zn	B	Cu	Mo
	0.01	0.005	0.002	0.002	0.002	0.006	0.001	0.003
**S-NM1**	73.84±0.15	10.79±0.21	85.02±0.02	3.83±0.03	4.84±0.03	11.01±0.01	1.12±0.03	0.62±0.01
**S-NM2**	102.36±2.35	29.95±0.05	80.79±0.78	7.23±0.03	10.28±0.06	10.88±0.12	2.13±0.02	0.59±0.01
**S-NM3**	109.29±2.71	39.65±0.35	123.51±0.48	14.09±0.08	20.59±0.12	17.39±0.39	2.28±0.03	0.91±0.01
**S-NM4**	182.59±1.69	71.47±0.46	218.18±0.82	17.98±0.02	15.15±0.09	22.71±0.29	2.76±0.03	0.83±0.01
**S-NX1**	280.76±2.24	97.72±0.28	237.71±0.29	15.37±0.37	19.52±0.11	27.72±0.28	2.07±0.04	0.90±0.01
**S-NX2**	193.43±3.42	60.94±0.06	129.90±0.09	14.81±0.19	18.35±0.11	19.21±0.21	2.26±0.05	0.74±0.00
**S-NX3**	188.55±2.17	94.43±0.28	139.57±0.33	18.17±0.16	17.67±0.09	28.76±0.78	2.45±0.05	0.75±0.01
**S-NX4**	178.82±1.17	92.29±0.88	108.67±0.33	13.16±0.16	16.74±0.09	29.78±0.21	2.53±0.06	0.85±0.00
**S-GS1**	195.86±1.13	94.90±0.09	134.30±0.30	23.36±0.35	18.52±0.10	24.39±0.39	2.83±0.06	0.77±0.01
**S-GS2**	305.56±4.56	132.38±0.62	256.41±0.40	31.67±0.67	19.16±0.11	32.11±0.10	4.28±0.06	0.97±0.02
**S-GS3**	331.92±2.08	158.91±0.08	269.36±0.63	38.25±0.25	22.82±0.13	38.54±0.54	4.83±0.06	1.02±0.02
**S-SX1**	272.36±2.63	91.02±1.02	140.24±0.75	25.40±0.39	21.55±0.12	22.78±0.21	3.55±0.08	0.80±0.00
**S-SX2**	281.37±3.63	101.45±1.45	115.36±0.35	26.71±0.29	13.96±0.08	23.61±0.60	2.53±0.09	0.66±0.00
**S-SX3**	262.93±2.92	115.50±0.50	236.75±0.75	39.46±0.45	12.23±0.07	28.88±0.11	3.40±0.11	0.90±0.01
**S-SHX1**	255.39±2.61	90.41±0.58	215.50±0.50	24.46±0.54	17.94±0.10	20.35±0.35	2.78±0.10	0.87±0.01
**S-SHX2**	268.17±1.83	85.17±0.16	218.49±1.50	20.61±0.60	18.22±0.11	21.05±0.05	2.67±0.10	0.83±0.01
**S-XJ1**	239.48±0.52	148.58±1.42	229.66±0.33	42.25±0.25	18.90±0.11	20.99±0.01	3.58±0.10	0.87±0.01
**I-GS1**	291.91±1.09	127.57±2.43	229.80±0.19	26.48±0.02	18.54±0.10	34.58±0.18	3.37±0.03	0.87±0.02
**I-GS2**	282.65±1.35	122.72±2.28	243.41±1.58	29.77±0.22	18.14±0.11	29.44±0.24	3.81±0.00	0.92±0.01
**I-GS3**	291.78±3.22	116.89±3.10	253.59±1.41	28.35±0.04	18.99±0.11	31.24±0.24	4.00±0.00	0.96±0.01
**I-GS4**	252.03±3.96	127.74±2.73	261.97±1.97	34.98±0.01	18.66±0.11	31.82±0.17	4.40±0.02	1.00±0.02
**I-GS5**	289.92±2.08	145.23±1.77	252.79±2.79	35.23±0.27	20.23±0.12	38.50±0.29	4.37±0.01	0.96±0.00
**I-GS6**	319.44±1.56	128.31±1.69	196.22±0.21	30.12±0.11	17.18±0.10	35.14±0.25	5.20±0.20	0.75±0.02
**I-GS7**	288.15±1.85	105.82±2.18	248.67±3.86	29.67±0.32	18.35±0.11	33.01±0.30	4.34±0.06	0.94±0.01
**I-GS8**	295.25±2.75	169.80±0.19	161.43±0.56	38.18±0.17	27.44±0.16	56.92±0.28	6.15±0.14	1.54±0.02
**I-GS9**	296.93±3.07	129.39±1.61	245.37±1.37	31.38±0.61	19.33±0.11	35.12±0.28	4.73±0.06	0.93±0.01
**I-GS10**	274.45±5.55	155.35±0.65	215.67±1.33	32.99±0.01	20.18±0.12	33.06±0.16	4.47±0.06	0.82±0.01
**I-GS11**	284.87±5.13	147.45±2.55	317.30±1.30	34.95±0.04	25.00±0.15	31.99±0.49	5.31±0.11	1.21±0.02
**I-GS12**	295.98±4.01	121.08±1.08	263.04±1.95	29.37±0.36	22.65±0.13	45.62±0.08	4.92±0.02	1.00±0.01
**I-GS13**	208.19±8.18	169.32±2.68	288.51±1.49	39.07±0.07	20.01±0.12	33.65±0.15	4.34±0.02	1.10±0.01
**I-NX1**	233.42±5.42	134.48±2.52	200.96±4.04	22.53±0.53	19.08±0.11	22.47±0.03	3.42±0.01	0.76±0.01
**I-NX2**	257.11±7.11	104.82±4.81	211.89±1.11	27.42±0.42	16.63±0.09	27.30±0.19	3.27±0.02	0.80±0.02
**I-NX3**	288.86±9.86	137.59±3.58	315.18±0.82	35.37±0.36	23.53±0.13	43.99±0.01	5.61±0.08	1.20±0.04
**P-XJ1**	261.18±4.17	119.31±1.69	252.96±2.04	23.12±0.11	16.38±0.09	25.52±0.08	2.60±0.01	0.96±0.03
**P-XJ2**	294.81±7.81	120.13±2.87	241.38±2.62	32.74±0.26	17.64±0.10	29.74±0.26	2.43±0.02	0.92±0.03
**P-XJ3**	225.03±19.97	110.08±1.08	168.38±1.62	28.53±0.46	17.45±0.10	23.70±0.30	3.21±0.02	0.96±0.01
**P-XJ4**	274.26±7.25	113.10±3.09	164.14±2.86	19.07±0.07	19.63±0.11	29.10±0.10	2.69±0.02	0.94±0.02
**P-XJ5**	282.96±6.95	198.74±1.25	173.41±1.58	34.71±0.29	24.32±0.14	26.80±0.19	4.69±0.11	1.13±0.03

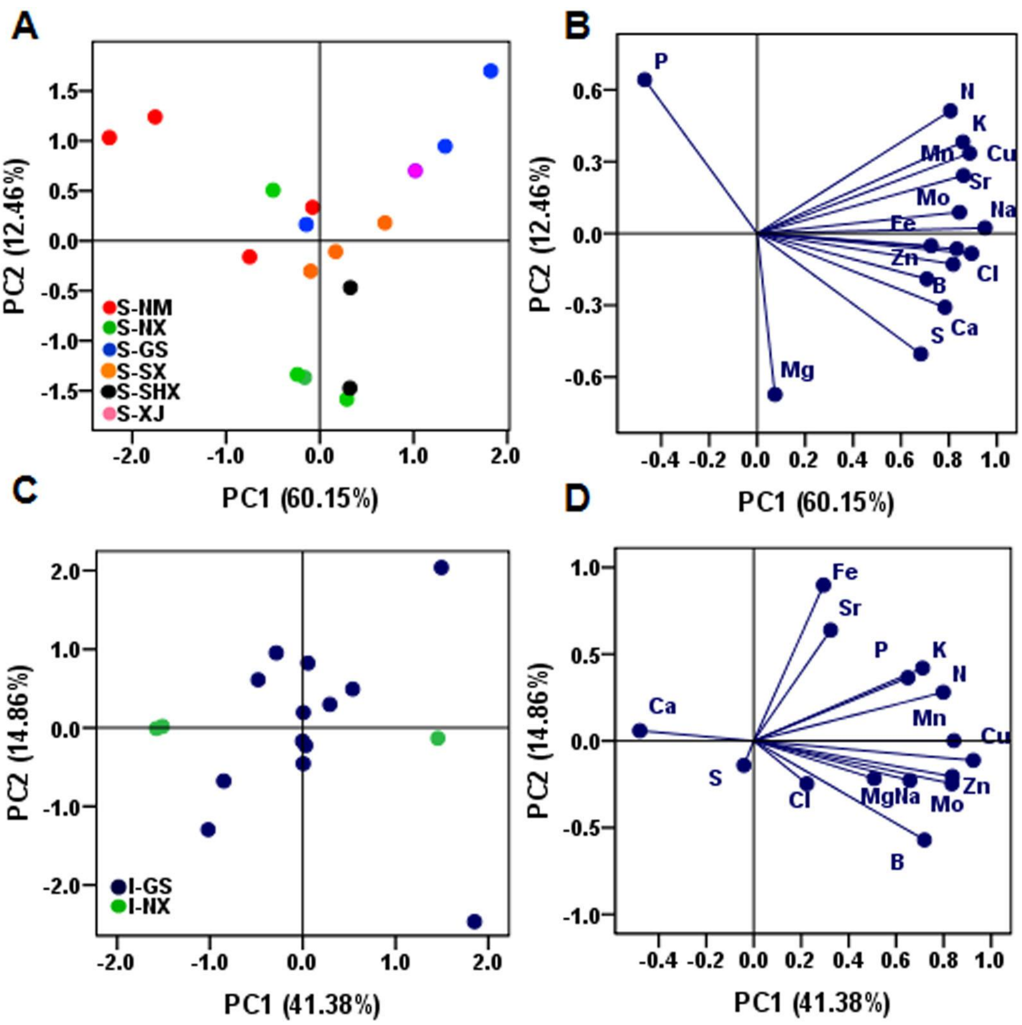

Fig. S1. Principal component analysis of *Ephedra* samples based on contents of 15 elements. A and B illustrate the score plot and the corresponding loading plot of 17 *E. sinica* samples from different provinces, respectively. Grouping according to geographical location was shown by PC1 and PC2, which explained 60.15% and 12.46% of the variance, respectively. C and D show the score plot and the corresponding loading plot of 16 *E. intermedia* samples from different provinces. Grouping according to geographical location was shown by PC1 and PC2, which explained 41.38% and 14.86% of the variance, respectively.

Table S2 Samples source of *Ephedra* in the present work.

Code	Coordinates	Locations	province	Code	Coordinates	Locations	province
**S-NM1**	E120° 37′ N43° 08′	Naimanqi		**I-GS4**	E105° 62′ N34° 98′	Qinan	
**S-NM2**	E119° 35′ N43° 52′	Balinzuoqi	Inner Mongolia	**I-GS5**	E106° 39′ N35° 33′	Kongdong	
**S-NM3**	E118° 59′ N42° 58′	Wulan		**I-GS6**	E103° 40′ N36° 08′	Annigbao	
**S-NM4**	E107° 30′ N38° 29′	Etuokeqi		**I-GS7**	E106° 03′ N35° 41′	Zhuanglang	
**S-NX1**	E107° 23′ N37° 47′	Yanchi		**I-GS8**	E101° 97′ N38° 24′	Yongchang	Gansu
**S-NX2**	E106° 24′ N37° 53′	Lingwu	Ningxia	**I-GS9**	E103° 72′ N36° 10′	Anning	
**S-NX3**	E106° 15′ N38° 17′	Yongning		**I-GS10**	E104° 38′ N35° 00′	Longxi	
**S-NX4**	E106° 09′ N38° 21′	Qingtongxia		**I-GS11**	E104° 53′ N34° 41′	Wushan	
**S-GS1**	E103° 06′ N37° 37′	Gulang		**I-GS12**	E103° 01′ N36° 97′	Tianzhu	
**S-GS2**	E108° 00′ N36° 44′	Huachi	Gansu	**I-GS13**	E107° 37′ N35° 23′	Jingchuan	
**S-GS3**	E107° 39′ N35° 31′	Qingyang		**I-NX1**	E106° 27′ N36° 19′	Guyuan	
**S-SX1**	E111° 53′ N39° 27′	Youyu		**I-NX2**	E106° 49′ N35° 52′	Pengyang	Ningxia
**S-SX2**	E113° 25′ N40° 08′	Datong	Shanxi	**I-NX3**	E106° 08′ N35° 37′	Longde	
**S-SX3**	E113° 54′ N40° 16′	Tianzhen		**P-XJ1**	E86° 16′ N42° 29′	Baluntai	
**S-SHX1**	E109° 77′ N34° 90′	Pucheng	Shaanxi	**P-XJ2**	E88° 29′ N42° 18′	Tulufan	
**S-SHX2**	E109° 72′ N34° 92′	Dali		**P-XJ3**	E86° 21′ N42° 19′	Hejing	Sinkiang
**S-XJ1**	E87° 10′ N42° 15′	Quhui	Sinkiang	**P-XJ4**	E86° 54′ N42° 17′	Heshuo	
**I-GS1**	E104° 29′ N35° 24′	Huining		**P-XJ5**	E91° 34′ N43° 22′	Hami	
**I-GS2**	E107° 54′ N35° 31′	Ningxian	Gansu				
**I-GS3**	E102° 88′ N37° 96′	Weiwu					

S: *E. sinica*, I: *E. intermedia,* P: *E. przewalskii*

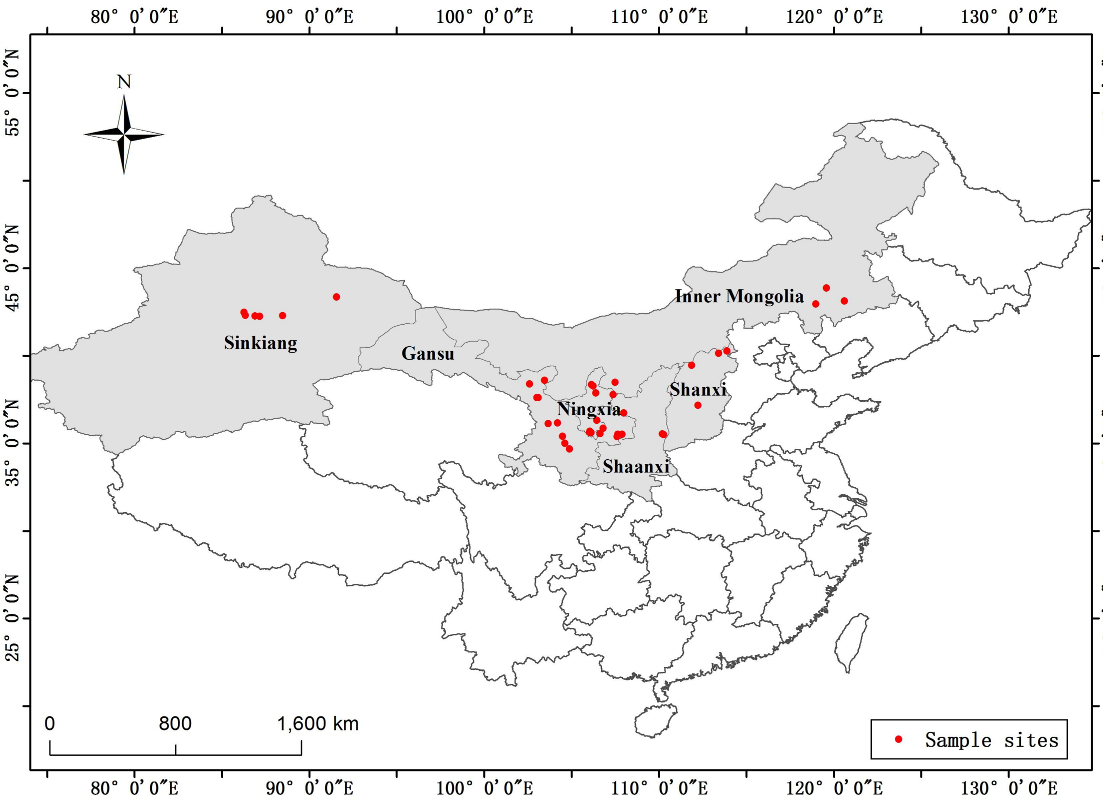

Fig. S2. Geographical origin locations of 38 *Ephedra* samples involved in six provinces for this study. Maps genera ted using ArcGIS 10.3.

(ArcMap URL: http://www.esri.com/en/arcgis/products/arcgis-pro/resources/arcmap-resources)
